# How Interviewees Determine What Interviewers Want to Know

**DOI:** 10.5334/irsp.1284

**Published:** 2026-06-04

**Authors:** David A. Neequaye, Alexandra Lorson, Holly K. Barnett

**Affiliations:** 1 Department of Psychology, Lancaster University, United Kingdom; 2 Department of Criminology, Mid Sweden University, Holmgatan 10, 85233, Sweden; 3 Center for Language and Cognition (CLCG), University of Groningen, The Netherlands

**Keywords:** disclosure, investigative interviewing, pragmatic correspondence, programmatic research

## Abstract

We examine the mechanisms by which interviewees in investigative interviews mentally organize information when deciphering what an interviewer wants to know. The overarching idea is that such a process stems from the extent to which an interviewer’s question specifies an objective. Our initial test (i.e., Neequaye & Lorson, 2023) suggested two competing mechanisms: High-specificity questions lead interviewees to focus on particularly relevant details to the exclusion of other information, while low-specificity questions make interviewees focus on a broader range of information items (Mechanism-1)—versus—Interviewees generally assume that interviewers want to know all the information at their disposal, irrespective of question specificity (Mechanism-2). We conducted two conceptual replications to gain clarity (Replication 1, *N* = 318; Replication 2, *N* = 292). The results were similar across the board. The more specific the questions an interviewer posed, the more likely interviewees homed in on the details that should provide a pragmatic response to those questions. And interviewees’ disposition, whether cooperative, semi-cooperative, or resistant, had no effect on information-item designations. Contrary to our expectations, interviewees remained confident that they had identified what their interviewer wanted to know, irrespective of Question-Specificity. This result held irrespective of whether the interviewer mixed high- and low-specificity questions (Replication 1) or consistently asked high- versus low-specificity questions (Replication 2). Thus, at this point in the research program, we lean more toward Mechanism-1.

Investigative interviews are formal social interactions wherein interviewers solicit information from interviewees in service of various issues (e.g., Neequaye, 2023). Such concerns include enhancing eyewitnesses’ ability to recall information, detecting lies, intelligence gathering, and insurance claims investigations (e.g., Fisher & Geiselman, 1992; Granhag & Hartwig, 2015; Hartwig et al., 2014; Warren & Schweitzer, 2018). Those domains of research concentrate on approaches interviewers use to elicit information. A fundamental assumption of this focus is that interviewees hypothesize about what their interviewers want to know. Interviewees must identify their interviewer’s objectives before they can determine the extent to which they might cooperate with or resist their interviewer’s requests. For example, one of the primary goals of the Scharff interviewing technique is to conceal the interviewer’s objectives so that it is not apparent to interviewees that they are contributing to those objectives (see, e.g., Oleszkiewicz, 2016). Conversely, the Cognitive Interview encourages interviewers to make their objectives explicit and use mnemonic devices to enhance interviewees’ recall of accurate information (Fisher & Geiselman, 1992). *But how do interviewees discern what their interviewers want to know?* This vital step in the investigative interviewing process has received little attention. The literature needs an explanation of the mechanisms by which interviewees flag their interviewer’s objectives. Then, researchers can better specify how various interviewing methods might exert their effects.

Theorists in Pragmatics have argued that when people converse, they piece together each other’s utterances to decode the messages being conveyed (e.g., Grice, 1989; McHoul, 1987). People pay more attention to utterances they believe will contribute a worthwhile difference to understanding the message being conveyed, and such perceived worthwhile utterances play a greater role in determining individuals’ comprehension of messages (Sperber & Wilson, 1987; Sperber & Wilson, 1995). And, following Gricean maxims, people respond with messages that are relevant to the perceived subject(s) of discussion (Grice, 1975).

Drawing on the Pragmatics literature (i.e., Grice, 1975; McHoul, 1987; Sperber & Wilson, 1995), we proposed that interviewees determine their interviewer’s objectives based on the extent to which an interviewer’s question specifies an objective (see Neequaye & Lorson, 2023, for contextual details and the basis of the initial theory). We contended that high-specificity questions that clearly indicate what an interviewer wants to know would lead interviewees to *mentally* flag information items that pragmatically correspond to those objectives. By *pragmatically correspond*, we mean information items that, *if uttered*, will objectively provide the information an interviewer’s question requests—not necessarily more or less information. Low-specificity questions wherein an interviewer’s objectives appear broad will introduce more uncertainty regarding what the interviewer wants to know. By asking high- or low-specificity questions,^1^ interviewers can influence the extent to which interviewees might discern what they want to know. In our initial proposal (i.e., Neequaye & Lorson, 2023), we limited the present theory to intelligence gathering. Here, we abandon that restriction and propose that the mechanisms apply to investigative interviews in general.

The following illustration provides an overview of the theory (see Figure 1). Imagine that multiple interviewees held the same body of information on a topic. Our contention is that the way an interviewer frames a question could lead those respective interviewees to focus on different aspects of that information corpus. High-specificity questions influence interviewees to focus on *particular* details (i.e., pragmatic correspondence) such that they ignore information that does not provide *the specific answer* a question requests. Conversely, low-specificity questions will make interviewees focus on a broader range of information items, given the uncertainty of the interviewer’s objectives. That is to say, how interviewers formulate questions influences what interviewees hone in on as the subject(s) of interest: this honing process can indirectly affect whether an interviewee cooperates or resists—wittingly or unwittingly. One must understand a question’s purpose before disposition or other contextual factors most proximate to disclosure can take effect. Identifying the question’s purpose has an indirect influence because it tells the cooperator or resistor how best to achieve their goals. The possibility of making interviewees witting or unwitting cooperators or resisters has crucial ethical and efficacy implications. We will revisit that issue later in the Discussion Section after our proposal has undergone the necessary testing.

**Figure 1:**
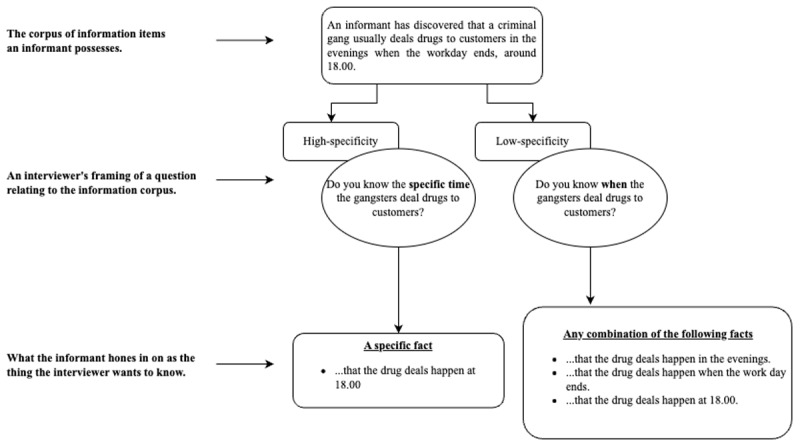
Proposed Mechanism Illustrating how Interviewees Determine what Interviewers Want to Know.

It is worth highlighting the distinction between the current research and related work, like memory activation and reporting (e.g., Koriat & Goldsmith, 1996). Theories on memory deal with what makes people (e.g., interviewees) report accurate or inaccurate information. The present research takes a step back to examine how interviewees determine what a question is questioning or asking for. This aspect of a conversation is a pragmatic matter, *yet to be* an issue of memory. One must first engage in some pragmatics—or decipher the objectives of a question—before turning to their memories to craft an answer (see Neequaye & Lorson, 2023 for an extended discussion). Consider the following example. An interviewee could accurately flag that the interviewer wants to know about a bomb design (pragmatic issue), but the interviewee may fail to remember the design because they did not encode that information when they previously encountered the bomb (memory issue). An unfortunate interaction between pragmatics and memory is worth mentioning here. It is possible for an interviewer to assume that their objective is clear, and an interviewee might confirm the interviewer’s assumption. However, that confirmation could be mistaken for reluctance or uncooperativeness if the interviewee cannot answer the interviewer’s question due to memory issues (e.g., encoding failure or forgetfulness).^2^ This possible conflict between pragmatic assumptions and memory highlights the need to better understand how interviewees determine what interviewers want to know. Then, researchers can advise practitioners that an interviewee acknowledging their information objectives does not automatically imply that the interviewee possesses the requisite information.

## Recapping an Initial Examination of the Theory: Neequaye and Lorson (2023)

Initial tests suggested that the process by which interviewees hone in on information items might differ from the original theory just described (i.e., Figure 1). We (i.e., Neequaye & Lorson, 2023) found that interviewees assume their interviewer wants to know *all* the information they hold on a subject under discussion. Most interviewees indicated that their interviewer wanted to know everything regardless of whether the interviewer posed a high- or low-specificity question (Neequaye & Lorson, 2023). However, interviewees were more confident that they had identified what their interviewer wanted to know when the interviewer posed high- versus low-specificity questions. We believe our previous procedure (i.e., Neequaye & Lorson, 2023, Study 1) raises issues worth addressing.

Neequaye and Lorson (2023) invited participants to act as informants in a criminal investigation. The relevant experiment (Study 1) comprised ten scenarios where participants discovered information about a gang under investigation. In each scenario, an interviewer posed a question to elicit information about the respective discoveries. The research employed a within-subjects design during the Question-Specificity trials. High-specificity questions always asked for the *complete details* participants held, while low-specificity questions were broad because they requested to know *any detail* about a discovery. Participants indicated what they thought their interviewer wanted to know via a predefined response list with three options: their discovery’s bare minimum, medium, or complete details. The mixture of high- and low-specificity questions plus the use of a predefined response list might have introduced a potential confound.

All things being equal, any investigator would want the complete details an interviewee holds, given that complete information would be more beneficial to any investigation than partial details. By mixing Question-Specificity (i.e., within-subject trials), it is possible that participants disregarded the uncertain nature of low-specificity questions. They might have inferred that their interviewer always wanted to know complete details because that same interviewer also frequently asked for complete details—the de facto purpose of any investigative interview. Moreover, Neequaye and Lorson’s (2023) design could have made such an assumption even more salient by presenting participants with a predefined list including complete details as a choice option. Taken together, Neequaye and Lorson’s (2023) design may have influenced participants to *always* hone in on complete details.

A further limitation of Neequaye and Lorson’s (2023) procedure (Study 1) is that pragmatic correspondence was designed to be equivalent to complete details such that complete details pragmatically corresponded to high-specificity questions. Put differently, high-specificity questions always asked for complete details in any given scenario (Neequaye & Lorson, 2023, Study 1). However, pragmatic correspondence does not necessarily denote complete or partial information. Pragmatic correspondence refers to *specific information* that, all things being equal, will truthfully answer an interviewer’s question; suppose the interviewee holds such information. As noted, Neequaye and Lorson’s (2023) protocol might have influenced interviewees to assume their interviewer always wanted to know complete details, regardless of question specificity. And because the experiment also equalized the status of pragmatic correspondence and complete details, the study may have failed to test whether high- versus low-specificity questions, indeed, draw attention to specific details.

## The Present Research

The argument that Neequaye and Lorson’s (2023) procedure influenced participants to always hone in on complete details is a speculation that remains to be verified. Here, we reexamine Neequaye and Lorson’s (2023) original theory while addressing the contentions we raised about their procedure. The present research included two replications of Neequaye and Lorson (2023, Study 1). The findings will assist us in determining further appropriate studies to uncover the mechanisms by which interviewees determine what interviewers want to know.

Similar to Neequaye and Lorson (2023, Study 1), the protocol of Replications 1 and 2 aimed to equalize the status of pragmatic correspondence and complete details. But Replication 1 implemented a within-subjects design for Question-Specificity trials, and Replication 2 employed a between-subjects design. Furthermore, in both replications, participants indicated what their interviewer wanted to know using a free text response rather than choosing from a predefined list.

Suppose Replications 1 and 2 generate Neequaye and Lorson’s (2023, Study 1) finding that interviewees focus on complete details, regardless of Question-Specificity, then Neequaye and Lorson’s (2023) original theory requires a significant revision. Such results would suggest that Question-Specificity has little influence on determining what an interviewer wants to know; interviewees likely assume that interviewers always want to elicit complete details. If Replication 1 replicates Neequaye and Lorson (2023, Study 1), but Replication 2 fails to replicate, then it is likely that the mixing of high- and low-specificity questions makes interviewees focus on complete details, which is not necessarily a de facto assumption that the purpose of any interview is to elicit complete details.

## Overview of Research Protocols and Hypotheses

In both studies, participants began by assuming the role of an informant whom an investigator has propositioned to inform on a drug-dealing gang. The informant role was manipulated so that participants would take on one of the following dispositions: cooperative, semi-cooperative, or resistant when engaging with the investigator. The informants made several discoveries about the gang’s operations, and the investigator asked them about those discoveries. Then, the informant role became an interviewee role, and the investigator became an interviewer. Some of the interviewer’s questions indicated an explicit objective (high-specificity), and the aim of others was comparatively uncertain (low-specificity). Interviewees did *not* give direct answers to the questions. The instructions invited them to (1) indicate what they think their interviewer wants to know and (2) give a confidence rating on that choice. The overarching goal of the research design is to ascertain whether high- versus low-specificity questions influence interviewees to focus on specific information (i.e., pragmatic correspondence). Suppose Neequaye and Lorson’s (2023) original proposal has significant verisimilitude; the subsequent predictions should receive support.

When interviewees determine what an interviewer wants to know, high-versus low-specificity questions should elicit more designations of information items aligning with pragmatic correspondence (Core Hypothesis 1). That is, high- versus low-specificity questions should significantly influence the perceived specificity of interviewees’ responses. Low-versus high-specificity questions should make interviewees less confident that they have identified what their interviewer wants to know (Core Hypothesis 2). This confidence assessment is a proxy to examine whether low- versus high-specificity questions make it challenging to determine what an interviewer wants to know. Disposition should have no effect on what interviewees think their interviewer wants to know (Core Hypothesis 3).

Neequaye and Lorson’s (2023) original theory should be revised if the following pattern of results emerges rather than support the Core Hypotheses. Question-specificity has no effect when interviewees determine what an interviewer wants to know (Revision Hypothesis 1). Question-specificity has no effect on interviewees’ confidence that they have flagged what their interviewer wants to know (Revision Hypothesis 2). Such findings will support the idea that interviewees assume their interviewer always wants to know all the information they possess. This potential revision to Neequaye and Lorson’s (2023) theory is a key aspect of the present research.

## Method: Replications 1 and 2

### Sampling Plan and Power Analysis

We conducted simulations to examine the level of precision the chosen sample size for each Replication Study (*n* = 300; total *N* = 600) can provide, given our resources and planned hypotheses tests. The simulations indicated that the planned sample size will suffice (see the Analysis Plan). Hence, we configured the sampling process in Prolific® to continue until at least 600 participants passed all attention checks and the IMC.

We recruited English-speaking participants aged ≥ 18 with an approval rating > 90% (compensation: £9/hr). The sampling process, which was beyond our control after the project launched, captured 638 prospective participants. Two participants did not give consent to participate and were excluded from further participation; 27 participants failed the instructional manipulation check (IMC); and one participant did not respond to the actual trials in the study. We remained with 610 participants in the end. Replication 1, which employed a within-subjects design for Question-Specificity, included 318 participants, and Replication 2, which employed a between-subjects design for Question-Specificity, included 292 participants. The majority of participants specified their gender as female, 220 as male, 7 as non-binary/third gender, and 8 people preferred not to disclose their gender or to specify their gender themselves. Most of the participants spoke either British English (*n* = 417) or American English (*n* = 120), with a few speaking Irish English or New Zealand English.

### Procedure

We describe the protocols of the replications at once for the sake of conciseness. When necessary, we highlight the differences between the protocols. The project received ethics approval (FST-2023-4117-RECR-4) at Lancaster University. The replications were conducted simultaneously to ensure that prospective participants did not partake in more than one experiment. The research was conducted entirely online via Qualtrics and presented as studies on communication within a law enforcement context.

The procedure protocol can be reproduced using the Qualtrics (qsf) file available here https://osf.io/qt4p3/.

**Phase 1: Informant Role**. Participants read a background story to assume the role of an informant who can gather information about a drug-dealing gang. The plot was structured so that informants faced the possibility of disclosing information to an interviewer, which is typical in investigative-interviewing research (e.g., Oleszkiewicz, 2016). The story manipulated informants’ dispositions by inviting them to take on either a *cooperative, semi-cooperative*, or *resistant* mindset when engaging with their interviewer (see Appendix A).

**Phase 2: Introduction to Decision-making Instructions**. Next, participants underwent an instruction stage to get acquainted with how to engage with the interviewer’s questions. An instructional manipulation check (IMC) was included to identify and exclude inattentive participants who failed the check (see Appendix B). The instructions informed participants that they would undergo a number of scenarios. In each scenario, they would discover information about the criminal gang being investigated. Then, they would receive a question from their interviewer about the earlier discovery. Participants were told to write what they think their interviewer *wants to know*—not what they intend to disclose.

**Phase 3: Discoveries, Questions, and Decision-making**. The format and number of scenarios for the Question-Specificity trials depended on the respective replications. Appendix C outlines the stimulus material in detail.

***Replication 1.*** Participants underwent six scenarios in random order. In each scenario, they discovered something about the gang under investigation. Similar to Neequaye and Lorson, (2023), the discoveries were such that participants could describe them in three legitimate ways: (*i*) bare minimum details; (*ii*) medium details—i.e., a new detail plus the bare minimum; or (*iii*) complete details—i.e., new detail plus the bare minimum and medium details. Consider this discovery: an informant overheard a phone call in which a gang member told a colleague, ‘It is better to sell the off-brand green-star oxycodone.’ A substantive description of that discovery could embody any of the following contents: they sell *oxycodone* (bare minimum), *green-star oxycodone* (medium), or *an off-brand green-star oxycodone* (complete).

Replication 1 employed a within-subjects design for the Question-Specificity trials. After each discovery, the interviewer asked a high- or low-specificity question, three questions per condition—participants underwent six trials. Following Neequaye and Lorson’s (2023) design, high-specificity questions *specifically* requested the complete details of a discovery. Low-specificity questions asked for *anything* such that participants could reasonably think the interviewer wanted to know the bare minimum, medium, or complete discovery.

**Figure 2:**
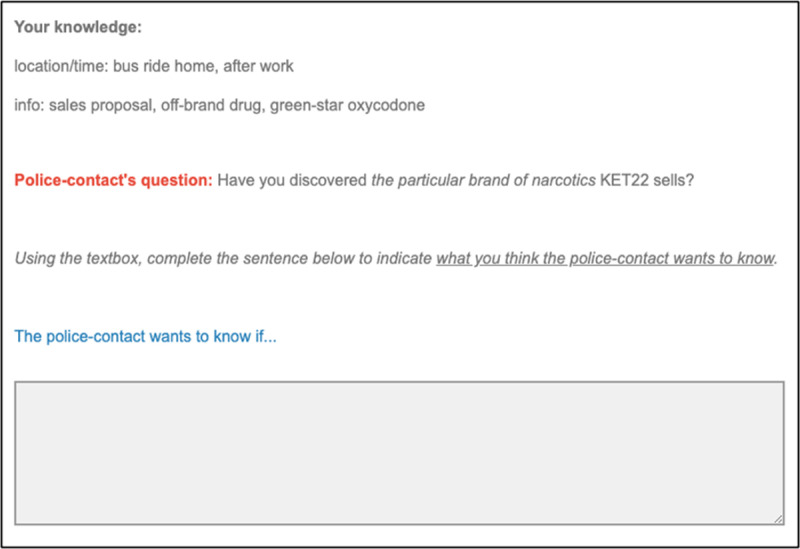
Illustration of Decision-making Page (with a High-specificity Question).

Next, participants were invited to write what they think their interviewer wants to know. Participants received a summary of their discovery—to ensure that they do not forget the contents, given that the present research is **not** about memory. The summary was followed by a textbox structured in a format to keep participants cognizant of the instruction to write what they think their interviewer’s objective is. The textbox was preceded by the prompt ‘The police-contact wants to know if…’ (see Figure 2). We settled on this protocol after conducting two pilot tests to determine how best to comprehensively capture participants’ thinking (see https://osf.io/rvmn6/).

After participants indicated what they thought the interviewer wanted to know, they provided two confidence ratings regarding their choice. One rating was mandatory: On a scale from 1–5, how confident are you that ‘*participants verbatim text*’ is what the police-contact wants to know (1 = *not confident at all*, 5 = *completely confident*)? The optional rating invited participants to place a hypothetical wager on whether their choice was what the interviewer wanted to know. The wager was a percentage of their compensation (0% = *none of my compensation*, 100% = *all of my compensation*). Given that the wager was optional, participants could decide to skip it. This setup provides two extra measures of confidence besides the mandatory confidence rating: (i) whether a participant is confident enough to place a wager and (ii) the extent of confidence as evident in a wager’s magnitude.

***Replication 2.*** This study employed a similar protocol as Replication 1 but used a between-subjects design for the Question-Specificity trials. Participants underwent five randomized scenarios in which the interviewer consistently asked either high- or low-specificity questions. Participants were randomly assigned to the high- or low-specificity condition.

#### Exclusion criteria

Both studies included four control questions to flag the data of inattentive participants (see Appendix D). The control questions were randomly distributed among the scenarios in each study. As noted, we excluded the data from participants who failed the IMC (see Appendix B) and those who failed one control question.

## Coding Strategy

We planned for two assistants, blind to the hypotheses, to code all participants’ responses. The coders received a data file containing anonymized participant IDs along with their responses and the corresponding scenarios.

**Figure 3:**
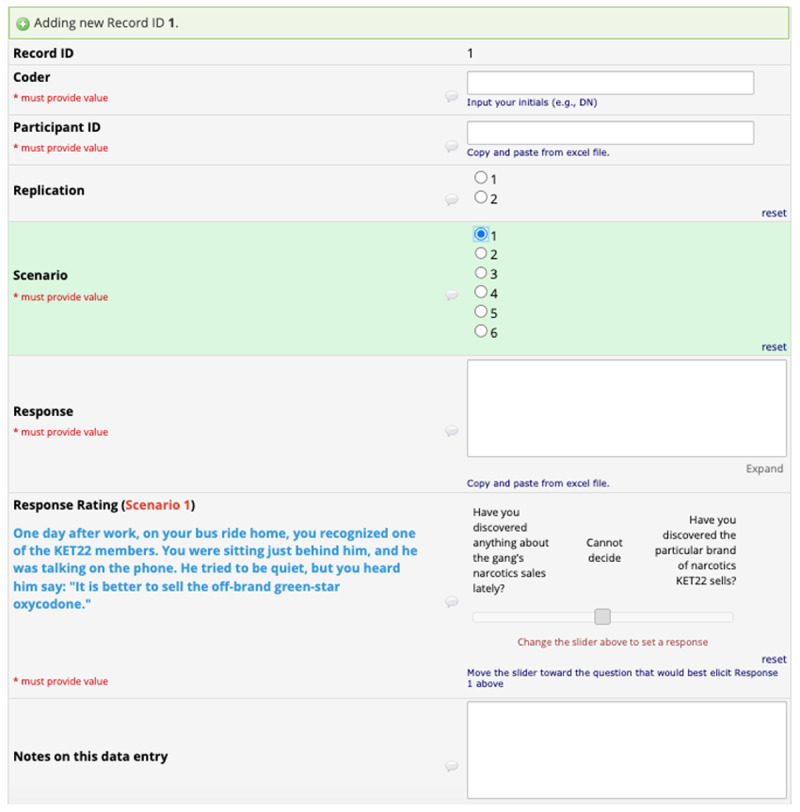
Illustration of codebook.

The assistants used the data file to execute their coding in a separate data entry form developed with REDCap (Research Electronic Data Capture), a secure web-based software platform hosted at Lancaster University. The coders first entered a participant’s ID and indicated the specific scenario being coded. The scenario selection revealed the contents of that scenario. The coders then entered, from the data file, the participant’s response regarding the scenario. This extra step is to ensure that the coders remain aware of the response undergoing coding. Next, they rated the extent to which the high- or low-specificity better elicited (or better fit) the response being coded. This rating was provided using a visual analog slider ranging from –100 to +100, including a zero (0) midpoint. The leftmost hand of the scale (i.e., –100) displayed the low-specificity question, and the rightmost hand (i.e., +100) displayed the high-specificity question. In this way, moving the slider to the left or right coded whether a participant’s response pragmatically corresponds more to the high- or low-specificity question. The descriptive label of the zero (0) mid-point was ‘cannot decide,’ and was used in the following situations: (1) when the response does not describe the contents of the scenario; (2) when the response says ‘no’ or ‘I do not know’; or (3) ‘when the response fits neither the high- or low-specificity question.’ Figure 3 depicts the coding, and the following link https://osf.io/krybn/ is where the code book (i.e., data dictionary) can be accessed.

### Interrater Reliability

Initially, we planned for each coder to code 30% of the data for an interrater reliability assessment before testing the hypotheses. We deviated from that plan because one of the coders became otherwise engaged and could not continue with the project. Consequently, the two coders independently coded a random 20% of the data for consistency assessment using the intraclass correlation coefficient (ICC). We preregistered that the minimum acceptable ICC was to be 0.6; if any scenario fell below this value, the coders would discuss the causes of disagreements and resolve them independently. Then, they would re-code the initial data until they achieve the minimum threshold. The initial consistency assessment met our criterion for acceptance in all six scenarios, with ICCs ranging from .75 to .90 (all *p*-values < .001). At the following link https://osf.io/32485/, one can verify the ICC ratings and all the coding can be accessed.

In our preregistration, we planned for the two coders to code all responses, from which we intended to create an index of pragmatic correspondence based on the average rating of both coders. As noted, however, one of our coders could not continue; given the acceptability of the consistency ratings, one coder coded the remaining data. We used their coding in the primary analysis. Furthermore, the distributions of response ratings across scenarios were similar, rendering an analysis of individual scenarios superfluous (see the Supplemental Material).

## Analysis Plan

### Overview

All the analyses were conducted with the R software environment (The R Foundation, n.d.). We planned to test our hypotheses (i.e., pragmatic correspondence preference and confidence) using Bayesian linear regression models via the brms package version 2.21 (Bürkner, 2017), which provides an interface to fit Bayesian mixed models via Stan (Stan Development Team, 2017). Tables X1 through X6 summarize the entire analysis strategy. Our contingency plan was that, if our models prove unsuitable given the data, we would employ alternative analysis approaches, such as the beta regression model. This contingency was to be determined using posterior predictive simulations (i.e., the brms function pp_check(), with ndraws = 100). Importantly, our decision regarding the data-generating process we assume to underlie the data would *not* be influenced by the hypothesis.^3^

Each analysis would produce posterior distributions over parameters, quantifying the probability of each possible parameter value, given the data. We planned to report the posterior mean with the corresponding 95% credible interval (95%-CrI) and the 95% highest density interval (HDI). The 95%-CrI is the range around the posterior mean within which the true value of the parameter lies with a probability of .95. The HDI is identical to the CrI if the posterior is symmetric; if the posterior is asymmetric, the endpoints of both intervals may differ.

Following best practices (Kruschke, 2014; Kruschke & Liddell, 2018; Vasishth et al., 2018), we defined a region of practical equivalence (ROPE) to examine whether the evidence that emerges from the findings is consistent with our predictions. The ROPE can be understood as a null region or a region encompassing parameter values that correspond to ‘no effect (given our theoretical propositions)’: effect sizes too small to be considered as supporting the hypotheses. We planned to assume that an effect warrants consideration for our theoretical propositions if the corresponding parameter’s HDI falls outside the null region. In other words, if the 95% HDI falls outside the ROPE, it means that the 95% most credible values of that parameter are not practically equivalent to the null region (Kruschke, 2014). If a parameter’s HDI overlaps with the null region and the sign is positive, we would reject a theory postulating a negative effect. We planned to reject a theory postulating a positive effect if the sign is negative. If the parameter’s HDI falls entirely within the null region, we would conclude that the data are consistent with ‘no effect’ (not to say that we have proved that the null hypothesis is true). That instance means that the 95% most credible values of the parameter are practically equivalent to the null region (Kruschke, 2014). And we would not settle on a conclusion from our data when the ROPE lies entirely within the parameter’s HDI.

Our resources allowed a sample size of approximately 600 participants (*N* = 300, per experiment). Given that constraint and our planned hypotheses tests, we conducted simulations to examine the level of precision the chosen sample size can provide. Our desired level of precision is that the width of parameter coefficients’ 95% HDIs should be equal to or smaller than 16 (based on Neequaye & Lorson, 2023). We ran four models with simulated data, which indicated that a model with 270 participants could reach our desired precision (https://osf.io/s5q7m/). The simulations did not include the within-participant correlational structure between responses in the high-specificity and low-specificity conditions and therefore approximate a between-participant structure rather than the within-participant design of the study. This approach constitutes a conservative simplification, as incorporating within-participant correlations would generally increase statistical power and thus reduce the required sample size.

### Model Specification: Pragmatic Correspondence

#### Fixed and Random Effects

To predict pragmatic correspondence preference, we planned to fit three truncated (lower bound –100 and upper bound = 100) Bayesian linear regression models, one per experiment (i.e., Models 1a and 2a) and one meta-analysis model (i.e., Model 3). The modes were to be truncated to account for the specific range of our dependent variable (i.e., [–100, 100]) since predictions outside this range are meaningless. Models 1a and 2a would include the same fixed effects but different random effects structures. The two models would include the variables disposition (cooperative vs. semi-cooperative vs. resistant) and question type (high- vs. low-specificity) as predictors. We planned to add the interaction of both predictors for exploratory purposes. To examine Core Hypothesis 1/Revision Hypothesis 1a, for both models, the predictors were sum-coded (question type: high-specificity = 1, low-specificity = –1; disposition: cooperative = 1, 0; resistant = 0, 1, semi-cooperative = –1, –1). To test Core Hypothesis 3, the disposition variable was treatment-coded with the cooperative condition as the reference level. Model 1a (Replication 1) included random by-item slopes for question type and disposition, together with random by-participant slopes for question type. Conversely, Model 2a (Replication 2) did not include random by-participant slopes for question type since Replication 2 employed a between-subjects manipulation for question type.

Model 3 targeted Revision Hypothesis 1b and tested whether the manipulation of question type—either as a within- or between-subjects factor—indeed influences the participants’ responses. To examine the effect of design type, the model included the predictors design type, question type, and disposition, plus the interaction of design type and question type. The model included random by-participant intercepts and random by-items slopes for design type, question type, disposition, and the interaction of design type and question type.

#### Priors

For all models, we planned to use the same weakly regularizing priors, allowing a reasonably wide range of parameter values. The priors for the intercept were normal distributions with mean 0 and standard deviation 20 based on the assumption that, averaged over question type (and for semi-cooperative participants), the perceived specificity should be centered around zero. Skepticism in this assumption is introduced by defining a relatively broad prior of 30. For fixed effects, normal priors with a mean of 0 and a standard deviation of 20 will be used. This prior is conservative because it assigns most probability mass to values close to zero, which would correspond to a null effect. Such an approach demonstrates our commitment to giving the Revision Hypothesis a worthwhile chance of receiving support. Random effects will be modeled as a correlation matrix and a vector of standard deviations. The standard deviations will be assigned half-normal priors with a mean of 0 and a standard deviation of 1. For the correlation matrix, an LKJ (2) prior will be used such that smaller correlations are favored over extreme values such as ±1 (Sorensen et al., 2016; Stan Development Team, 2017). We planned to carry out a prior-sensitivity analysis to assess whether priors dominate the posterior distribution.

#### Region of Practical Equivalence and Model Comparison

The specified ROPE of [–8, 8] was based on Neequaye and Lorson’s (2023) initial finding about the effect of low- versus high-specificity questions on participants’ decisions regarding what an interviewer wants to know (i.e., Neequaye & Lorson, 2023). Neequaye and Lorson’s (2023) results indicated that the lowest possible difference between the question type conditions was 4%. Applying that finding to the outcome variable of the present research (a continuous value ranging from –100 to 100), a 4% difference translates to a difference of 8 and a ROPE of [–8, 8]. We specified the same ROPE for the effect of disposition and the interaction terms. We planned that if the data did not fit with a normal distribution and called for a different model, we would consider a ROPE of [–0.17, 0.17] on the log-odds scale directly derived from Neequaye and Lorson (2023).

#### Sampling Process

We drew samples from the posterior distributions of the model parameters using the NUTS sampler (Hoffman & Gelman, 2014). Four sampling chains were run, each collecting 4,000 iterations. The first 1,000 iterations were disregarded as part of the warm-up phase, leaving 12,000 for analysis. By our estimations, this sampling process should be the same for all models, and the chains should mix well (all R = 1.0).

We planned that should our models show divergent transitions after warm-up, we would follow suggested solutions to resolve divergences that involve changing the MCMC criteria, for example: by raising adapt_delta, increasing the number of iterations, or increasing tree depth, etc. Suppose we run into convergence issues and this model formulation turns out to be impossible to estimate without divergent transitions, even after tweaking the MCMC criteria; we planned to assess whether dropping by-item or by-participant random slopes would achieve convergence.

#### Predictions

To support Core Hypothesis 1, we expected the following result: High-specificity questions should elicit a greater preference for pragmatic correspondence than low-specificity questions. The 95% HDI of the test parameter should fall completely outside the ROPE; and considering that the question type variable will be sum-coded (high-specificity = 1, low-specificity = –1), the test parameter’s 95% HDI should have a positive sign. If the parameter’s 95% HDI falls completely within the specified ROPE, we would interpret our findings as being consistent with Revision Hypothesis 1a. Furthermore, we would consider our findings consistent with Revision Hypothesis 1b, if for Model 3, the interaction parameter’s 95% HDI falls completely inside the specified ROPE. We would count this finding as evidence against the claim that high-specificity questions only elicit greater pragmatic correspondence if the interviewer consistently uses high-specificity questions (as mirrored by Replication 2’s between-subjects design).

According to Core Hypothesis 3, the disposition variable should have no effect on preference for pragmatic correspondence when determining what an interviewer wants to know. The 95% HDI of the test parameter should fall completely within the ROPE, such that the 95% most credible values are practically equivalent to what we consider to be negligible effect sizes.

### Model Specification: Confidence

#### Fixed and Random Effects

We planned to examine confidence ratings by fitting two mixed-effects Bayesian ordinal (cumulative) regression models (Models 1b and 2b). Question type and disposition were included as predictors. Model 1b (i.e., Replication 1) included varying intercepts and slopes for participants and items, assuming that the effect of question type, on confidence ratings, varies between participants and scenarios. Model 2b (i.e., Replication 2) included varying intercepts for participants as both disposition and question type were between-subjects factors.

#### Priors

We used the default priors of brms except for the priors of the intercept and main condition effect. We used weakly regularizing priors, which allow a reasonably wide range of parameter values while penalizing very extreme values. The prior for the intercept was normally distributed with mean 0 and standard deviation 1. Furthermore, to build mild skepticism into our models, we set a weakly informative prior on the condition effect (Lemoine, 2019; McElreath, 2016): a normal distribution centered at zero with a standard deviation of 0.5. The LKJ prior is set to 2. A prior-sensitivity analysis was conducted to assess whether priors are dominating the posterior distribution.

#### Prediction

Core Hypothesis 2 would receive support if low- versus high-specificity questions led interviewees to be less confident that they have flagged what their interviewer wants to know—that difference should emerge, regardless of disposition. The test parameter’s 95% HDI should fall completely outside the ROPE, and because the question type variable was sum-coded (high-specificity = 1, low-specificity = –1), the parameter’s 95% HDI should have a positive sign. If the parameter’s 95% HDI falls completely within the specified ROPE, we would interpret our findings as being consistent with Revision Hypothesis 2.

### Model Specification: Wager (i.e., Secondary Confidence Variable)

We included a second confidence measure to assess participants’ willingness to bet that their preference was what the interviewer wanted to know. Analogous to the confidence ratings, we predicted that low- versus high-specificity questions will lead to a lower probability of betting, independent of disposition. We planned to report the posterior mean, the 95% credible interval (95%-CrI), and the probability that a given coefficient is greater than zero, given the data and model. This analysis is exploratory.

#### Fixed, Random Effects, and Priors

We examined participants’ willingness to place a wager by fitting two mixed-effects Bayesian logistic regression models (Models 1c and 2c). Question type and disposition were included to predict wagers. Model 1c (i.e., Replication 1) included varying intercepts and slopes for participants and items, assuming that the effect of question type on confidence ratings varies between participants and scenarios. Model 2c (i.e., Replication 2) included varying intercepts for participants only because disposition and question type are between-subjects factors.

We employed the same prior structure as described for the primary confidence measure.

## Study Design Template

**Notes:**

1. The outcome measure for Models 1a and 2a is a continuous variable ranging from –100 to 100.
2. **Sampling plan and test sensitivity rationale:** We aimed to remain with a minimum of *N* = 600 participants, *N* = 300 per study. Resource constraints and previous research (to precisely estimate a Region of Practical Equivalence [ROPE]) determined our sample size choice. For Replications 1 and 2, it holds that we will not conclude anything from our data when the ROPE lies entirely within the parameter’s HDI.
3. **Theory that could be shown wrong by outcomes:** Our goal was to ascertain the verisimilitude of two competing mechanisms following Neequaye and Lorson’s findings (2023). *Mechanism-1*: High-specificity questions lead interviewees to focus on particularly relevant details to the exclusion of other information, while low-specificity questions make interviewees focus on a broader range of information items; versus, *Mechanism-2*: Interviewees generally assume that interviewers want to know all the information at their disposal, irrespective of question specificity. We cannot rule out plausible effects that are smaller than the limits of our ROPE. Nonetheless, supporting the respective core hypotheses will count as support for Mechanism-1, but rejecting the Core Hypothesis will count as support for Mechanism-2.

**Table X1:** Replication 1: Question Type as a Within-Subjects Factor.


HYPOTHESIS	MODEL 1A	ANALYSIS	PREDICTIONS

**Core Hypothesis 1** High- versus low-specificity questions should elicit more designations of information items that align with pragmatic correspondence	brm(Specificity | trunc(ub = 100, lb = –100) ∼ Disposition + QuType + (QuType | SubjectID) + (Disposition + QuType | Context) Contrast coding for Model 1a: **Question-type:** high-specificity = 1, low-specificity = –1 **Disposition:** cooperative = 0 1 resistant. = 1 0 semi-coop. = –1 –1 The model for Replication 1 will only include the two predictors Question Type and Disposition and no interaction term. A model including an interaction term will be run for exploratory purposes.	To test this hypothesis, we investigated whether there is a main effect of question-type on the perceived specificity of participants’ responses.	The Question Type parameter’s HDI should lie outside the ROPE and have a positive sign for high-specificity questions (which are coded as 1).

**Revision Hypothesis 1a** High- versus low-specificity questions do not elicit more designations of information items that align with pragmatic correspondence.	brm(Specificity | trunc(ub = 100, lb = –100) ∼ Disposition + QuType + (QuType | SubjectID) + (Disposition + QuType | Context) Contrast coding for Model 1a: **Question-type:** high-specificity = 1, low-specificity = –1 **Disposition:** cooperative = 0 1 resistant. = 1 0 semi-coop. = –1 –1	To test this hypothesis, we investigated whether there is a main effect of question-type on the perceived specificity of participants’ responses.	The Question Type parameter’s HDI is predicted to fall within the null region, such that we can conclude the data are consistent with ‘no effect’ of question-type (not to say that we have proven that the null hypothesis is true).

**Core Hypothesis 3** There should be no effect of disposition on preference for pragmatic correspondence.	brm(Specificity | trunc(ub = 100, lb = –100) ∼ Disposition + QuType + (QuType | SubjectID) + (Disposition + QuType | Context) Contrast coding for Model 1a: **Question-type:** high-specificity = 1, low-specificity = –1 **Disposition (treatment):** cooperative = 0 0 resistant = 1 0 semi-coop. = 0 1	To test this hypothesis, we investigated whether there is a main effect of Disposition on the perceived specificity of participants’ responses.	All the Disposition parameter’s HDIs are predicted to fall within the null region, such that we can conclude the data are consistent with ‘no effect’ of disposition (not to say that we have proven that the null hypothesis is true).


**Table X2:** Replication 2: Question Type as a Between-Subjects Factor.


HYPOTHESIS	MODEL 2A	ANALYSIS	PREDICTIONS

**Core Hypothesis 1** High-versus low-specificity questions should elicit more designations of information items that align with pragmatic correspondence	brm(Specificity | trunc(ub = 100, lb = –100) ∼ Disposition + QuType + (1 | SubjectID) + (Disposition + QuType | Context) Contrast coding for Model 2a: **Question-type:** high-specificity = 1, low-specificity = –1 **Disposition:** cooperative = 0 1 resistant. = 1 0 semi-coop. = –1 –1 The model for Study 2 will only include the two predictors Question Type and Disposition and no interaction term. A model including an interaction term will be run for exploratory purposes.	To test this hypothesis, we investigated whether there is a main effect of question-type on the perceived specificity of participants’ responses.	The Question Type parameter’s HDI should lie outside the ROPE and have a positive sign for high-specificity questions (which are coded as 1).

**Revision Hypothesis 1a** High-versus low-specificity questions do not elicit more designations of information items that align with pragmatic correspondence.	brm(Specificity | trunc(ub = 100, lb = –100) ∼ Disposition + QuType + (QuType | SubjectID) + (Disposition + QuType | Context) Contrast coding for Model 1a: **Question-type:** high-specificity = 1, low-specificity = –1 **Disposition:** cooperative = 0 1 resistant. = 1 0 semi-coop. = –1 –1	To test this hypothesis, we investigated whether there is a main effect of question-type on the perceived specificity of participants’ responses.	The Question Type parameter’s HDI is predicted to fall within the null region, such that we can conclude the data are consistent with ‘no effect’ of question-type (not to say that we have proven that the null hypothesis is true).

**Core hypotheses 3** There should be no effect of disposition on preference for pragmatic correspondence.	brm(Specificity | trunc(ub = 100, lb = –100) ∼ Disposition + QuType + (QuType | SubjectID) + (Disposition + QuType | Context) Contrast coding for Model 1a: **Question-type:** high-specificity = 1, low-specificity = –1 **Disposition (treatment):** cooperative = 0 0 resistant = 1 0 semi-coop. = 0 1	To test this hypothesis, we investigated whether there is a main effect of disposition on the perceived specificity of participants’ responses.	All the Disposition parameter’s HDIs are predicted to fall within the null region, such that we conclude that the data are consistent with ‘no effect’ of Disposition (not to say that we have proven that the null hypothesis is true).


**Table X3:** Investigating Question Type in Interaction with Design Type.


HYPOTHESIS	MODEL 1A	ANALYSIS	PREDICTIONS

**Revision Hypothesis 1b** High- versus low-specificity questions manipulated as a between-subjects versus within-subjects factor do not elicit more designations of information items that align with pragmatic correspondence.	brm(Specificity | trunc(ub = 100, lb = –100) ∼ Disposition + QuType + DesignType + QuType:DesignType + (1 | SubjectID) + (Disposition + QuType + DesignType + QuType:DesignType | Context) Contrast coding for Model 3: **Question-type:** high-specificity = 1, low-specificity = –1 **Disposition:** cooperative = 0 1 resistant. = 1 0 semi-coop. = –1 –1 **Design-type:** between = 1 within = –1	To test this hypothesis, we investigated whether there is an interaction effect of question-type and design-type on the perceived specificity of participants’ responses.	The interaction parameter’s HDI is predicted to fall within the null region, such that we can conclude the data are consistent with ‘no effect’ of question type x design type (not to say that we have proven that the null hypothesis is true).


**Confidence in decisions (Replication 1)**.

**Table X3:** Replication 1: Confidence Ratings.


HYPOTHESIS	MODEL 1B (ORDINAL CUMULATIVE MODEL)	ANALYSIS	PREDICTIONS

**Core hypotheses 2** High-versus low-specificity question should make participants more confident in their designation choices, independent of disposition.	brm(Confidence ∼ Disposition + QuType + (QuType | SubjectID) + (Disposition + QuType | Context) Contrast coding for Model 1b: **Question-type:** high-specificity = 1, low-specificity = –1 **Disposition (changes to treatment coding to assess disposition, see above):** cooperative = 0 1 resistant. = 1 0 semi-coop. = –1 –1 The model included the two predictors Question Type and Disposition and no interaction term. A model including an interaction term was run for exploratory purposes.	To test this hypothesis, we investigated whether there is a main effect of Question Type on the confidence ratings.	The Question Type parameter’s HDI should lie outside the ROPE and have a positive sign for high-specificity questions (which are coded as 1). The Disposition parameter’s HDI is predicted to fall within the null region, such that we conclude that the data are consistent with ‘no effect’ of Disposition on the confidence ratings (not to say that we have proven that the null hypothesis is true).


Output (5-point Likert Scale): ‘not confident at all,’ ‘slightly confident,’ ‘somewhat confident,’ ‘fairly confident,’ ‘completely confident’.

**Table X4:** Replication 1: Willingness to Bet (Exploratory).


HYPOTHESIS	MODEL 1C (ORDINAL CUMULATIVE MODEL)	ANALYSIS	PREDICTIONS

High- versus low-specificity questions should increase the probability of betting, independent of disposition.	brm(Bet ∼ Disposition + QuType + (QuType | SubjectID) + (Disposition + QuType | Context) Contrast coding for Model 1C: **Question-type:** high-specificity = 1, low-specificity = –1 **Disposition (changes to treatment coding to assess disposition, see above):** cooperative = 0 1 resistant. = 1 0 semi-coop. = –1 –1 The model included the two predictors Question Type and Disposition and no interaction term. A model including an interaction term was run for exploratory purposes.	To test this hypothesis, we investigated whether there is a main effect of Question Type on the confidence ratings.	The Question Type parameter’s HDI should lie outside the ROPE and should have a positive sign for high-specificity questions (which are coded as 1). The Disposition parameter’s HDI is predicted to fall within the null region, such that we conclude that the data are consistent with ‘no effect’ of Disposition on the confidence ratings (not to say that we have proven that the null hypothesis is true).


Output: Willingness to bet, ‘yes’ (1), ‘no’ (0).

**Confidence in Decisions (Replication 2)**.

**Table X5:** Replication 2: Confidence Ratings.


HYPOTHESIS	MODEL 2B (ORDINAL CUMULATIVE MODEL)	ANALYSIS	PREDICTIONS

**Core hypotheses 2** High- versus low-specificity question should make participants more confident in their designation choices, independent of disposition.	brm(Confidence ∼ Disposition + QuType + (1| SubjectID) + (Disposition + QuType | Context) Contrast coding for Model 2B: **Question-type:** high-specificity = 1, low-specificity = –1 **Disposition (changes to treatment coding to assess disposition, see above):** cooperative = 0 1 resistant. = 1 0 semi-coop. = –1 –1 The model included the two predictors Question Type and Disposition and no interaction term. A model including an interaction term was run for exploratory purposes.	To test this hypothesis, we investigated whether there was a main effect of question-type on the perceived specificity of the responses.	The Question Type parameter’s HDI should lie outside the ROPE and have a positive sign for high-specificity questions (which are coded as 1). The Disposition parameter’s HDI is predicted to fall within the null region, such that we conclude that the data are consistent with ‘no effect’ of Disposition on the confidence ratings (not to say that we have proven that the null hypothesis is true).


Output (5-point Likert Scale): ‘not confident at all,’ ‘slightly confident,’ ‘somewhat confident,’ ‘fairly confident,’ ‘completely confident’.

**Table X6:** Replication 2: Willingness to bet (exploratory).


HYPOTHESIS	MODEL 2C (ORDINAL CUMULATIVE MODEL)	ANALYSIS	PREDICTIONS

High- versus low-specificity questions should increase the probability of betting, independent of disposition.	brm(Bet ∼ Disposition + QuType + (1 | SubjectID) + (Disposition + QuType | Context) Contrast coding for Model 2C: **Question-type:** high-specificity = 1, low-specificity = –1 **Disposition (changes to treatment coding to assess disposition, see above):** cooperative = 0 1 resistant. = 1 0 semi-coop. = –1 –1 The model included the two predictors Question Type and Disposition and no interaction term. A model including an interaction term was run for exploratory purposes.	To test this hypothesis, we investigated whether there is a main effect of Question Type on the participants’ willingness to bet.	The Question Type parameter’s HDI should lie outside the ROPE and should have a positive sign for high-specificity questions (which are coded as 1). The Disposition parameter’s HDI is predicted to fall within the null region, such that we conclude that the data are consistent with ‘no effect’ of Disposition on the confidence ratings (not to say that we have proven that the null hypothesis is true).


Output: Willingness to bet, ‘yes’ (1), ‘no’ (0).

## Results

The data supporting the results, full analysis code, and supplemental material can be accessed here: https://osf.io/2vqwa/overview.

### Replication 1: The Influence of Question-Specificity and Disposition on Information-Item Designation

**Figure 4:**
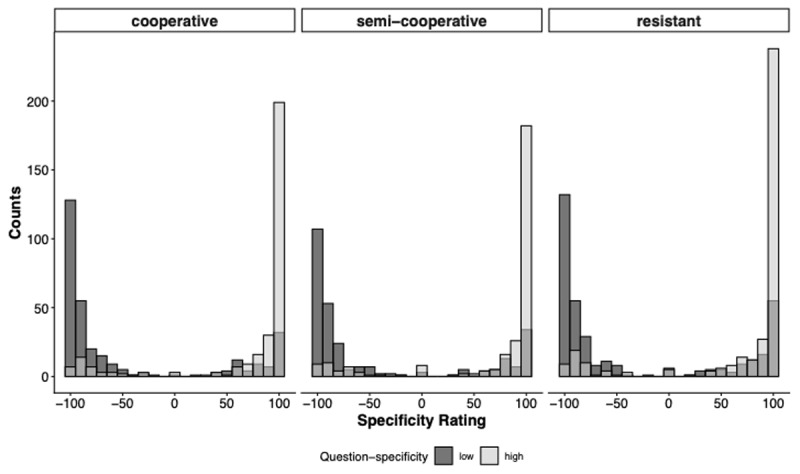
Histograms Showing the Distribution of Specificity Ratings by Question-Specificity Condition and Disposition. *Note*. Ratings are binned into 30 equally sized intervals per facet.

Participants’ responses regarding what their interviewer wanted to know covered the entire possible range; high-specificity (100) to low-specificity (–100). The overall mean was 13.64 (*Mdn* = 70, *Mo* = 100). As depicted in Figure 4, regardless of disposition, high-specificity questions elicited more specific information item designations than low-specificity questions.

As shown in Figure 4, the data are bimodal, with peaks around –100 and 100, rather than normally distributed. Accordingly, the preregistered linear regression model did not provide an optimal fit to the observed pattern. As noted in the preregistered analysis plan, we instead fitted a beta regression model. To do so, the Specificity Rating was transformed to fall within the interval (0, 1), excluding exact 0 and 1 values. Model comparison using leave-one-out cross-validation indicated that the preregistered main effects Model 1a did not perform worse than a model that included an interaction of both predictors, with a negligible difference in expected log predictive density (elpd diff = –1.5, se diff = 1.2). Accordingly, we focus our interpretation on the main effects model. We parametrized the beta distribution in terms of the parameters μ (mean) and ϕ (precision), in analogy to mean and variance for the normal distribution. This approach allowed us to examine whether question type and disposition influenced the mean estimates, while also accounting for variation in these effects across conditions.

Following Neequaye and Lorson (2023), we defined a region of practical equivalence (ROPE) of [–0.17, 0.17] on the logit scale for Question-Specificity effects. This approach corresponds to odds ratios between approximately 0.84 and 1.19 and reflects differences of limited substantive relevance. Accordingly, a 95% HDI width of 0.34 or smaller indicates that the posterior interval falls entirely within twice the ROPE range and therefore provides sufficient precision to detect or rule out effects of practical importance. With Model 1a, this level of precision was achieved for all coefficients.

**Figure 5:**
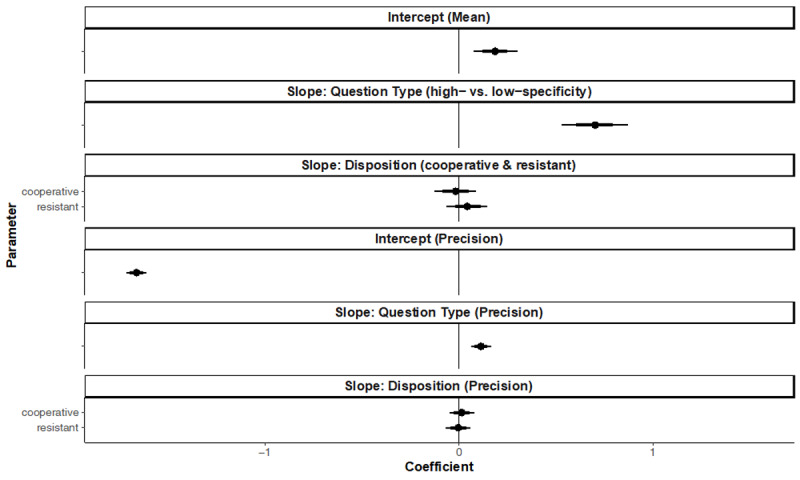
Posterior Distributions over Population-Level Estimates for Model 1a with 80% and 95%. *Note*. The highest density intervals and the ROPE area are shaded in light grey.

The desired level of precision (i.e., the width of the coefficients’ 95% HDIs should be equal to or smaller than 0.34) was reached for all coefficients in Model 1a. Focusing on Question-Specificity, Model 1a indicated that high- as opposed to low-specificity questions led to a reliable increase in specificity ratings (*b* = 0.70, HDI: [0.53, 0.87]), see Table 1. Since the coefficient’s HDI lies outside the ROPE and has a positive sign for high-specificity questions, we can confirm Core Hypothesis 1 and refute Revision Hypothesis 1. High- versus low-specificity questions elicited more specific information item designations (i.e., perceived specificity of what interviewees thought the interviewer wanted to know), see Figure 5 for an illustration.

**Table 1:** Population-Level Estimates of Model 1a in Log-Odds with the Standard Errors and 95% Highest Density Intervals.


PARAMETER	COEFFICIENT	POSTERIOR MEAN	Est. ERROR	l-95% HDI	u-95% HDI

μ	Intercept	0.19	0.06	0.07	0.30

μ	Question-Specificity (high)	0.70	0.09	0.52	0.86

μ	Disposition (cooperative)	–0.02	0.05	–0.12	0.09

μ	Disposition (resistant)	0.04	0.06	–0.07	0.15

ϕ	Intercept	–1.66	0.03	–1.72	–1.61

ϕ	Question-Specificity (high)	0.11	0.03	0.06	0.16

ϕ	Disposition (cooperative)	0.01	0.03	–0.05	0.08

ϕ	Disposition (resistant)	–0.01	0.03	–0.07	0.06


*Note*. Mean parameters are depicted first. The slope for Question-Specificity is the change in log-odds for the high-specificity question (1, high-specificity; –1, low-specificity) and the slope for disposition is the change in log-odds for cooperative and resistant participants (semi-cooperative was coded as –1, –1).

Furthermore, our model indicated no effect of Disposition, such that the HDIs of both Disposition coefficients fall within the null region, which means that our data is consistent with ‘no effect’ of Disposition on the level of designation specificity and Core Hypothesis 3. The effects of Question-Specificity and Disposition did not vary across conditions (ϕ estimates) as they lie entirely within our null region.

We pre-registered an additional model with a different contrast coding, which allowed us to compare the cooperative and resistant Disposition Conditions (with the cooperative condition as the reference level). The desired level of precision was not reached for the slope Disposition coefficients (95% HDI width of 0.38 and 0.37). Although this is slightly higher than our required precision, the Disposition coefficients still fall within the null region, indicating that our data are consistent with ‘no effect’ of Disposition on the level of designation specificity. The full model is available in Appendix E.

### Replication 1: Question-Specificity and Disposition’s Influence on Confidence

**Figure 6:**
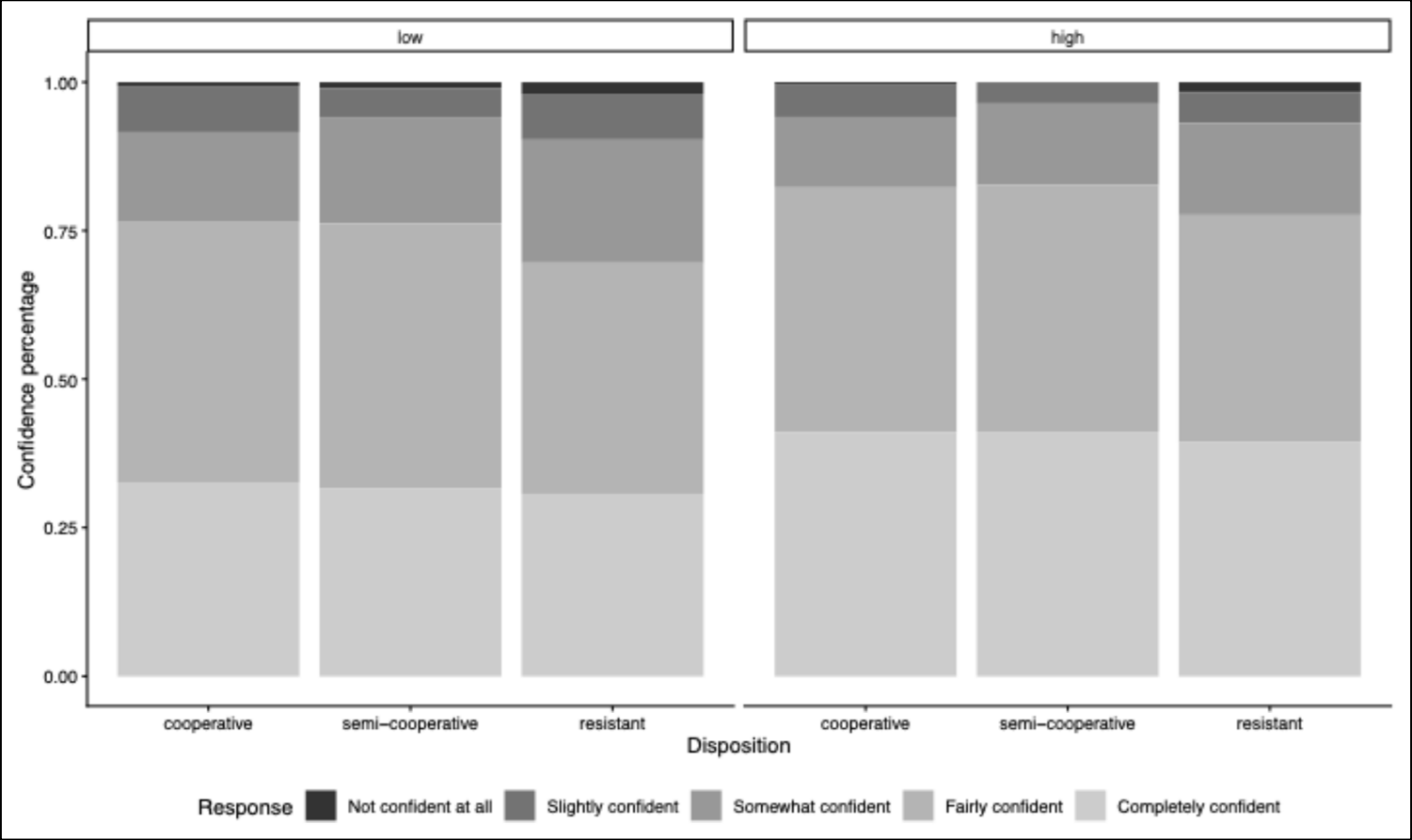
Relative Percentages of Confidence in Information Item Designations (Replication 1). *Note*. Distribution of confidence responses by disposition, shown separately for low- and high-specificity questions. Bars represent proportional response frequencies within each Disposition Condition.

Figure 6 indicates that, overall, participants were more confident in identifying what the interviewer wanted to know when the interviewer asked high- rather than low-specificity questions—indicated by more frequent ‘Completely confident’ and fewer ‘Not confident at all’ responses in the high- versus low-specificity condition. Participants in the resistant condition seemed slightly less confident than those in the semi-cooperative and cooperative conditions.

To examine Core Hypothesis 2—High- versus low-specificity questions should make participants more confident in their designation choices, independent of their disposition—we conducted an ordinal cumulative model (Model 1b). The model did not reveal any reliable effects, since all slope coefficients intersect or fall within the pre-defined region of practical equivalence.^4^ In addition, the slope coefficients of 95% HDIs did not achieve the necessary precision, indicating that we cannot draw strong conclusions from our data (see Table 2).

**Table 2:** Population-Level Estimates of Model 1b in Log-Odds with the Standard Errors and 95% Highest Density Intervals.


COEFFICIENT	POSTERIOR MEAN	Est. ERROR	l-95% HDI	u-95% HDI

Intercept [1]	–4.65	0.61	–5.91	–3.53

Intercept [2]	–2.38	0.59	–3.54	–1.21

Intercept [3]	–0.27	0.60	–1.51	0.83

Intercept [4]	2.83	0.61	1.52	3.92

Question-Specificity (high)	0.31	0.19	0.52	0.86

Disposition (cooperative)	0.07	0.22	–0.12	0.09

Disposition (resistant)	–0.19	0.20	–0.07	0.15


*Note*. The slope for Question-Specificity is the change in log-odds for the high-specificity question (1, high-specificity; –1, low-specificity) and the slope for disposition is the change in log-odds for cooperative and resistant participants (semi-cooperative was coded as –1, –1).

### Replication 2: The Influence of Question-Specificity and Disposition on Information-Item Designation

**Figure 7:**
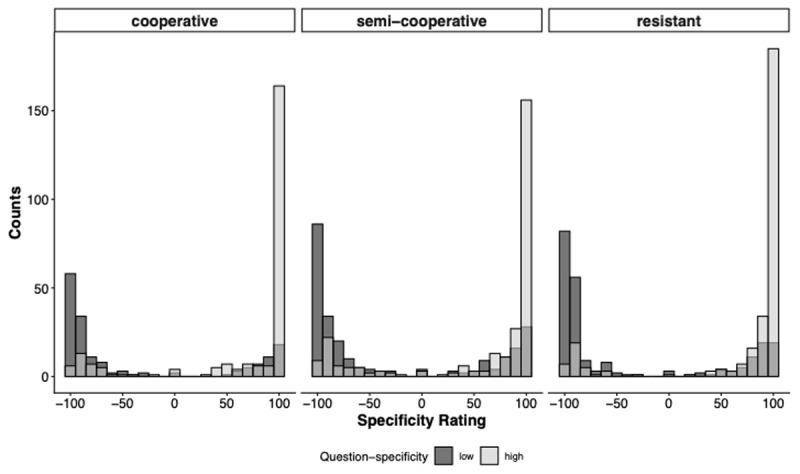
Histograms Showing the Distribution of Specificity Ratings by Question-Specificity Condition and Disposition. *Note*. Ratings are binned into 30 equally sized intervals per facet.

Similar to Replication 1, participants’ information item designations covered the entire possible range; high-specificity (100) to low-specificity (–100); *M* = 19.33, *Mdn* = 78, Mo = 100). As Figure 7 illustrates, regardless of disposition, high-specificity questions elicited more specific information item designations than low-specificity questions.

**Figure 8:**
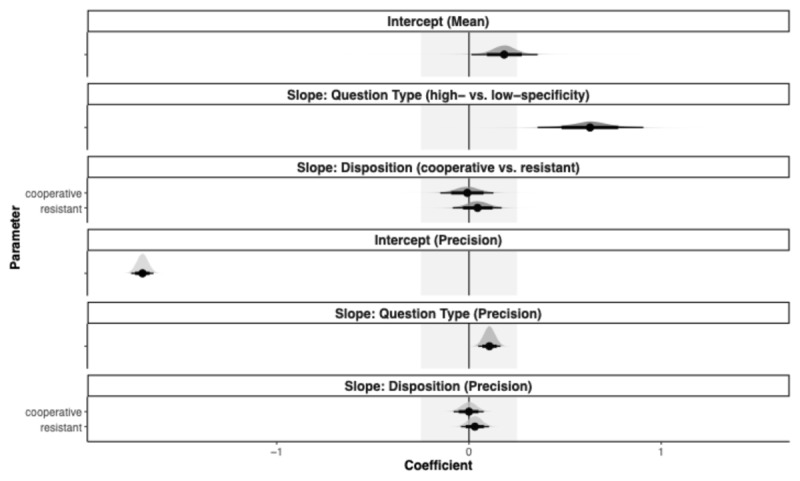
Posterior Distributions Over Population-Level Estimates for Model 2a with 80% and 95%. *Note*. The highest density intervals and the ROPE area are shaded in light grey.

As observed in Replication 1, the data distribution in Replication 2 is bimodal. We therefore ran beta regression models using the same analysis strategy as Model 1a. With Model 2a, the desired level of precision (i.e., the width of the coefficients’ 95% HDIs should be equal to or smaller than 0.34) was not achieved for the Question-Specificity slope coefficient. Model 2a indicated that high- rather than low-Specificity questions led to a reliable increase in the specificity of information item designations (*b* = 0.63, HDI: [0.34, 0.89]; see Table 3). Since the coefficient’s HDI lies outside the ROPE and has a positive sign for high-specificity questions, we can confirm Core Hypothesis 1 and refute Revision Hypothesis 1 (see Figure 8) for an illustration. Importantly, according to our preregistered decision criteria, the Revision Hypothesis could only have been conclusively refuted if the required level of precision had been achieved, thereby ruling out effects of practical relevance with sufficient certainty.

**Table 3:** Population-Level Estimates of Model 2a in Log-Odds with the Standard Errors and 95% Highest Density Intervals.


PARAMETER	COEFFICIENT	POSTERIOR MEAN	Est. ERROR	l-95% HDI	u-95% HDI

μ	Intercept	0.18	0.10	0.00	0.36

μ	Question-Specificity (high)	0.63	0.13	0.34	0.89

μ	Disposition cooperative	–0.01	0.08	–0.15	0.13

μ	Disposition resistant	0.05	0.07	–0.08	0.17

ϕ	Intercept	–1.70	0.03	–1.76	–1.64

ϕ	Question-Specificity	0.11	0.03	0.05	0.16

ϕ	Disposition cooperative	–0.00	0.04	–0.08	0.08

ϕ	Disposition resistant	0.03	0.04	–0.05	0.10


*Note*. Mean parameters are depicted first. The slope for Question-Specificity is the change in log-odds for the high-specificity question (1, high-specificity; –1, low-specificity), and the slope for Disposition is the change in log-odds for cooperative and resistant participants (semi-cooperative was coded as –1, –1).

Furthermore, our model revealed no effect of Disposition, such that the HDIs of both Disposition coefficients fall within the null region, indicating that our data is consistent with ‘no effect’ of Disposition on the level of designation specificity and Core Hypothesis 3. The effects of Question-Specificity and Disposition did not vary across conditions (ϕ estimates) as they fall entirely within our null region.

We pre-registered an additional model with a different contrast coding, which allowed us to compare the cooperative and resistant Disposition Conditions (with the cooperative condition as the reference level). The desired level of precision was not reached for any of the slope coefficients. Accordingly, the following results should be interpreted with caution and need further testing. The Disposition coefficients fall again within the null region, indicating that our data is consistent with ‘no effect’ of Disposition on the level of designation specificity. The full model is available in Appendix E.

### Replication 2: Question-Specificity and Disposition’s Influence on Confidence

**Figure 9:**
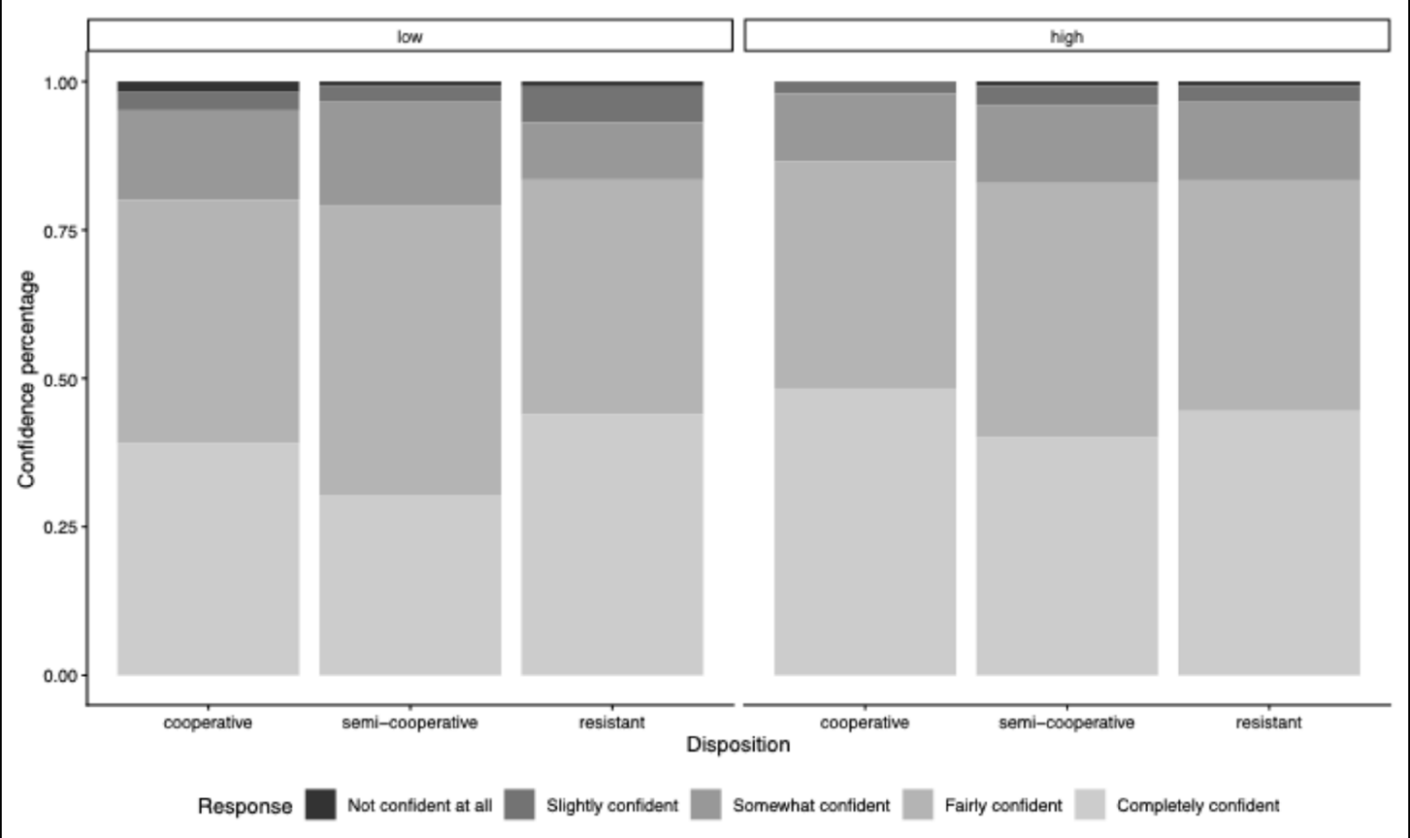
Relative Percentages of Confidence in Information Item Designations (Replication 2). *Note*. Distribution of confidence responses by disposition, shown separately for low- and high-specificity questions. Bars represent proportional response frequencies within each Disposition Condition.

Participants exposed to high- versus low-specificity questions were more confident in determining what the interviewer wanted to know—indicated by more frequent ‘Completely confident’ and fewer ‘Not confident at all’ responses in the high- versus low- Question-Specificity Conditions (see Figure 9). It is worth noting that the difference in confidence ratings is less pronounced in Replication 2 compared to Replication 1.

We ran a cumulative model (Model 2b) to formally test whether participants exposed to high- versus low-specificity questions are more confident in their designation choices, independent of their disposition. The model did not yield any reliable effects; all slope coefficients intersect or fall within the pre-defined region of practical equivalence. In addition, the 95% HDIs for the slope coefficients did not achieve the required precision. Thus, we cannot draw any strong conclusions from our data (see Table 4).

**Table 4:** Population-Level Estimates of Model 2b in Log-Odds with the Standard Errors and 95% Highest Density Intervals.


COEFFICIENT	POSTERIOR MEAN	Est. ERROR	l-95% HDI	u-95% HDI

Intercept [1]	–4.00	0.56	–5.09	–2.89

Intercept [2]	–2.26	0.53	–3.31	–1.26

Intercept [3]	–0.09	0.53	–1.09	0.96

Intercept [4]	3.23	0.54	2.19	4.30

Question-Specificity (high)	0.26	0.21	–0.16	0.67

Disposition (cooperative)	0.13	0.25	–0.39	0.60

Disposition (resistant)	0.13	0.25	–0.38	0.61


*Note*. The slope for Question-Specificity is the change in log-odds for the high-specificity question (1, high-specificity; –1, low-specificity) and the slope for disposition is the change in log-odds for cooperative and resistant participants (semi-cooperative was coded as –1, –1).

## General Discussion

Our goal was to examine how interviewees mentally organize information when deciphering what an interviewer wants to know. Previous research suggested two competing mechanisms (Neequaye & Lorson, 2023 [Study 1]). High-specificity questions lead interviewees to focus on particularly relevant details to the exclusion of other information, while low-specificity questions make interviewees focus on a broader range of information items (Mechanism-1)—versus—Interviewees generally assume that interviewers want to know *all* the information at their disposal, irrespective of Question-Specificity (Mechanism-2).

We conducted two conceptual replications of Neequaye and Lorson (2023) to gain clarity. The results were highly similar across the board. High-specificity questions elicited more specific information-item designations than low-specificity questions did. The more specific the questions an interviewer posed, the more likely interviewees homed in on the details that should provide a pragmatic response to those questions. And interviewees’ disposition, whether cooperative, semi-cooperative, or resistant, had no effect on information-item designations. It is worth noting that this result held irrespective of whether the interviewer mixed high- and low-specificity questions (Replication 1) or consistently asked high- versus low-specificity questions (Replication 2). Thus, at this point in the research program, we lean more toward Neequaye and Lorson’s (2023) original theory (i.e., Mechanism 1). High-specificity questions lead interviewees to mentally flag information items that pragmatically correspond to the question’s stated objectives; low-specificity questions keep a broader range of information items in play.

Contrary to our expectations, the analysis examining the influence of Question-Specificity on confidence did not achieve the desired precision in both studies. We cannot make any claims about whether low- versus high-specificity questions make interviewees less confident in identifying what an interviewer wants to know. Our best speculation, at this time, is that the imprecision might be due to the research design. Participants self-generated the information items rather than choosing from a predefined list (i.e., Neequaye & Lorson, 2023). People prefer self-consistent over discrepant feedback (Szumowska et al., 2022). Such a self-verification effect may have resulted in high confidence in predicting the information items the interviewer was asking for, especially in the low-specificity conditions. Again, this is a speculation that remains to be verified. Further research should explore whether our assumption that Question-Specificity influences confidence in identifying the interviewer’s objectives is warranted. Given that the present study and Neequaye and Lorson (2023) have not quite established a clear link between Question-Specificity and confidence, we are now skeptical of confidence as a proxy measure of how interviewees mentally organize information.

### Limitations and Future Directions

A potential limitation of the current research is that it is difficult to verify whether participants actually engaged in the meta-cognitive tasks when reporting their mental designations of information items. However, we included an instructional manipulation check (IMC) to ensure that participants were aware of the instructions, and there were control questions to ensure that participants were reading the stimulus material. Our pilot tests and the current data give us little reason to doubt that participants heeded the instructions. Importantly, the data and coding process of this research is completely open, allowing an independent reanalysis. That notwithstanding, the current research program will benefit from an alternative research design that makes reporting information-item designations consequential. For example, future research could have participants play the role of a double-agent informant. Their task would be to communicate the interviewer’s objective to a handler (without revealing the questions). And the design could be arranged such that errors in communicating what the interviewer wants to know would attract penalties. In this way, the study can include an in-built incentive to focus on the meta-cognitive processes of information-item designation.

### Concluding Remarks

The pragmatics of forensic investigative interviews have received little attention. But this aspect of the process could have significant downstream consequences. In this work, we demonstrate that Question-Specificity acts as a honing device, guiding interviewees on what information can be used to assist or resist an interviewer. These findings suggest that Question-Specificity is an essential ingredient that can actively make the interview more, or less transparent. The more transparent an interviewer’s objectives, the more opportunity is afforded the interviewee in choosing what to disclose.

## Additional File

The additional file for this article can be found as follows:

10.5334/irsp.1284.s1 Appendices. Appendix A to F.

## Peer Community In Registered Reports

**Figure:**
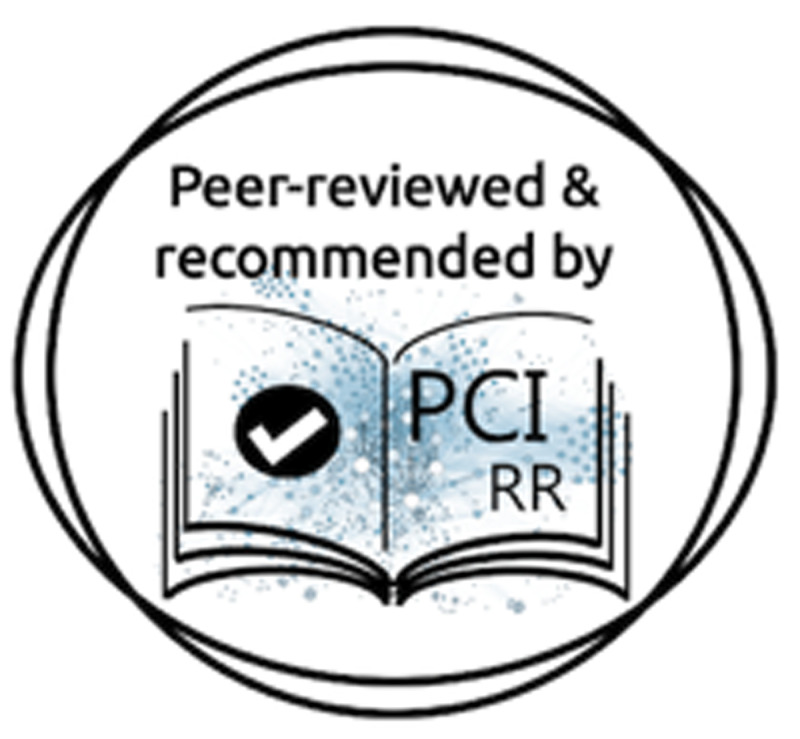


Stage 1 IPA (PCI RR): https://rr.peercommunityin.org/articles/rec?id=843.

Stage 2 recommendation (PCI RR): https://rr.peercommunityin.org/articles/rec?id=1914.

## Data Availability

All data supporting the findings in this research are publicly available here: https://osf.io/jb3y6/.

## Footnotes

In previous work, we used the phrases high-worthwhileness and low-worthwhileness questions. Now, we use the phrases high-specificity and low-specificity, as *specificity* is a more intuitive suffix than *worthwhileness*.

Many thanks to Feni Kontogianni for drawing our attention to this issue.

We planned to use another model if the predictive draws deviate from our data, indicating that a revised approach to testing the hypothesis is warranted. Such a decision cannot be determined objectively until the actual analysis: we might encounter a bimodal distribution, making it challenging to run our models under the assumption of a normal distribution. An example of being influenced by the hypothesis is that a normal distribution underlies our data. Then, we use linear regression, but after failing to support the hypothesis, we change to a beta model to fish for effects despite the linear regression model being the appropriate tool. We planned *not* implement such questionable practices.

The analysis regarding wagers on participants’ confidence that they had identified what the interviewer wanted to know revealed the same pattern of results, as did Replication 2. Those findings (Replication 1 & 2) are reported in Appendix F.
