# Supplementary material for: How Interviewees Determine What Interviewers Want to Know
Source: Int Rev Soc Psychol. 2026 Jun 4;39:10. doi: 10.5334/irsp.1284 (PMC13239355; doi:10.5334/irsp.1284)
Supplement: Appendices. — Appendix A to F. [file irsp-39-1284-s1.pdf]

Appendices

Introduction

This study is about communication within a law enforcement context. You will read some fictional scenarios assuming the role of the main character. Then you will answer some questions about each scenario. **Most of this study involves reading. So, please read the scenarios and instructions carefully because understanding them is crucial**. We have included questions to check if you read and answered questions with your full attention.

The entire study will take approximately 10 - 15 minutes to complete. You will receive a compensation of £2.25 for participating once the study is over.

Appendix A

Disposition manipulations

Imagine that you are one of the owners of a restaurant in town; you also work at this restaurant, which overlooks a big park. You and your colleagues have a good picture of what goes on in the park. It is well known among the restaurant staff that a narcotics-dealing gang called KET22 operates in the park. Recently, a police-contact approached you and your colleagues to provide information about the gang if you discovered anything. The police-contact mentioned that none of you are obliged to give any information. <**Dispositional variations begin here**>

Cooperative: However, KET22 disrupts your business at the restaurant. So, it is in your best interest to assist the police in their investigations to eliminate the gang. Then, your business can grow.

Semi-cooperative: KET22 disrupts your business at the restaurant. Assisting the police in their investigations could eliminate the gang. Then, your business can grow. But KET22 might retaliate if they find out you are helping the police take them down. So, you must strike a good balance between assisting the police and safeguarding yourself.

Resistant: The police-contact does not know this, but because you (personally) came into some financial troubles, you occasionally supply narcotics to customers at the restaurant on the gang's behalf. If the gang gets busted, you are very likely to get in trouble, too. You only agreed to meet with the police-contact to avoid suspicion.

Manipulation check (disposition)

Now you know your character or the role you are to play in this study. Suppose you were to make discoveries that could get KET22 busted, and the police-contact asked you about those discoveries. How would you engage with the interviewer?

- I will lie to ensure that I hide what I know. (-1)
- I will keep silent and not respond to the question. (0)
- I will reveal some of my discoveries, not everything I know. (1)
- I will reveal what I know. (2)

Appendix B

Introduction to scenarios

In the next phase of the study, you will be placed in various scenarios where you will make various discoveries about KET22, the gang under investigation. After each discovery, you will receive a question from the police contact about the discovery.

Your task in the upcoming phase is to **indicate what you think the police-contact WANTS TO KNOW about your discovery based on the police-contact's question**. The task is NOT about indicating what you will necessarily say in response to the question.

Your task is to indicate what you think the police-contact wants to know based on the police-contact's question!

We have included other questions to check if you read and answered the questions with your full attention.

Instructional manipulation check

What is **TRUE about your main task** in the upcoming phase?

- My task is to indicate what I think the police-contact wants to know based on the police-contact's question. **[Pass]**
- My task is to indicate what I want to say in response to the police-contact's question. **[Fail]**

Next follows the scenarios.

Appendix C

Replication 1

To facilitate randomization, participants will be randomly assigned to one of two lists. The lists' contents will be presented in random order in both the utterance and designation conditions.

	List 1	List 2
Scenario	1a	1b
Scenario	2b	2a
Scenario	3a	3b
Scenario	4b	4a
Scenario	5a	5b
Scenario	6b	6a

Scenarios - Replication 1

a. High-specificity questions are highlighted in green.

b. Low-specificity questions are highlighted in yellow.

1. One day after work, on your bus ride home, you recognized one of the KET22 members. You were sitting just behind him, and he was talking on the phone. He tried to be quiet, but you heard him say: "It is better to sell the off-brand green-star oxycodone."
    a. Have you discovered *the particular brand of narcotics* KET22 sells?
    b. Have you discovered anything about the gang's narcotics sales lately?
2. You always come to work earlier than your colleagues because you supervise the cleaners. You've realized that the KET22 gangsters usually arrive shortly after you in a blue Nissan Qashqai. By paying more attention, you've memorized the license plate number: FBT038.
    a. Do you know *the full details about the vehicle the KET22* gangsters usually arrive in at the park?
    b. Do you have any information about KET22's transportation in the park?

3. Lately, you have noticed a particular spot at the park where the KET22 gangsters deal drugs in the evenings. The spot is one of the park's exits, EXIT 7F. All the exits are located at different edges of the park, but 7F is rather discreet.

   a. Have you spotted *the exact location* at the park where KET22 deals drugs?
   b. Have you spotted anything about where KET22 deals drugs?

4. On your way home after work, you saw that some KET22 gangsters were arguing. It was around 19.00 on Monday. From what you heard, the argument was about whether to sell a high dose of drugs to a VIP customer.
   a. Have you caught *the contents of particular interactions* between the gang members lately?
   b. Have there been any developments with the gang members lately?

5. At work last week, your colleague, who is becoming friends with a KET22 gangster, slipped you some details. She said that KET22 is connected to a much bigger gang called TETO. TETO supplies opioids wholesale.
   a. Do you have information about the *sources from which KET22 obtains narcotics*?
   b. Has anything about KET22's narcotics operations come to your attention?

6. During one of your short breaks at work, you decided to enjoy some sunshine. So, you went to the edge of the park where there are benches. As you approached, you saw a rowdy group at one of the benches, and you chose the bench furthest away from them. The group was talking about how to contact KET22 to buy narcotics. They said customers could make contact by sending a text message containing a lion emoji to any KET22 phone number.
   a. Have you made observations about *exactly how customers contact* KET22 to buy narcotics?
   b. Have you made any observations about KET22's customers?

Replication 2

This study will employ a between-subjects design.

Scenarios - Replication 2

a. High-specificity questions are highlighted in green.

b. Low-specificity questions are highlighted in yellow.

1. One day after work, on your bus ride home, you recognized one of the KET22 members. You were sitting just behind him, and he was talking on the phone. He tried to be quiet, but you heard him say: "It is better to sell the off-brand green-star oxycodone."
   a. Have you discovered *the particular brand of narcotics* KET22 sells?
   b. Have you discovered anything about the gang's narcotics sales lately?

2. You always come to work earlier than your colleagues because you supervise the cleaners. You've realized that the KET22 gangsters usually arrive shortly

after you in a blue Nissan Qashqai. By paying more attention, you've memorized the license plate number: FBT038.

   a. Do you know *the full details about the vehicle the KET22* gangsters usually arrive in at the park?
   b. Do you have any information about KET22's transportation in the park?

3. Lately, you have noticed a particular spot at the park where the KET22 gangsters deal drugs in the evenings. The spot is one of the park's exits, EXIT 7F. All the exits are located at different edges of the park, but 7F is rather discreet.
   a. Have you spotted *the exact location* at the park where KET22 deals drugs?
   b. Have you spotted anything about where KET22 deals drugs?

4. On your way home after work, you saw that some KET22 gangsters were arguing. It was around 19.00 on Monday. From what you heard, the argument was about whether to sell a high dose of drugs to a VIP customer.
   a. Have you caught *the contents of particular interactions* between the gang members lately?
   b. Have there been any developments with the gang members lately?

5. At work last week, your colleague, who is becoming friends with a KET22 gangster, slipped you some details. She said that KET22 is connected to a much bigger gang called TETO. TETO supplies opioids wholesale.
   a. Do you have information about the *sources from which KET22 obtains narcotics*?
   b. Has anything about KET22's narcotics operations come to your attention?

Confidence rating (After each scenario)

- The police-contact asked:

  [*display question*]

- Based on the above question, **you wrote**: the police-contact wants to know if

  [*display selection*]

  - On a scale from 1 – 5, how confident are you that **what you wrote** the police-contact wants to know?

    1 = *not confident at all*, 2 = *slightly confident*, 3 = *somewhat confident*, 4 = *fairly confident*, 5 = *completely confident*

Confidence measure via bets [optional question]

- Suppose you were to **place a bet on your text below**.
- **You wrote** the police contact wants to know if [*display text*]
- On a scale from 0 to 100, what percentage of your compensation (for participating in this research) are you willing to bet that **what you wrote** is what the police-contact wants to know.

  0% = none of my compensation, 100% = all of my compensation

<u>Appendix D</u>

Control Questions

The control questions will employ the same scenario outlined below. The scenario will be presented four times, in random order, with four different questions.

Recently, a man came into the restaurant to buy coffee. You suspect he might be one of the KET22 gangsters, but you are unsure. When he made his order, there was no milk at the counter. So, you asked your colleague to get some milk from the fridge in the back. While you were waiting, you got a good look at his face and stature. You can guess that he is about 190cm tall. His hair was dark with grey streaks. He had green eyes and a scar on his left jaw. The name on the card he used to pay for his drink was Kari Jupo.

Q1. From the options below, select the name on the card the man used to pay for his drink.

- Minea Blankson
- Johnny Biles
- Kari Jupo
- Renave Olsson

Q2. From the options below, select the correct description of the man's hair.

- Blonde with brown streaks
- Blonde with grey streaks
- Dark with grey streaks
- Dark with yellow streaks

Q3. From the options below, select what the man ordered.

- Sandwich
- Coffee
- Beer
- Salad

Q4. From the options below, select the correct description of the man's height

- 190cm
- 200cm
- 164cm
- 175cm

Appendix E

Additional analyses

Investigating Question-Specificity in interaction with Design Type

We investigated whether high-versus low-specificity questions manipulated as a between- versus within-subjects factor did not elicit more designations of information items that align with pragmatic correspondence, i.e. high Specificity Ratings (Revision hypothesis 1b). To test this hypothesis, we ran a Beta regression model and focused on whether there was a reliable interaction effect of Question-Specificity and Design-Type. Our model did not disclose a reliable interaction effect, in that the

interaction parameter's 95% HDI falls within the null-region consistent with "no effect", see table Z1 for the full model output.

Parameter	Coefficient	Posterior mean	Est. error	l-95% HDI	u-95% HDI
μ	Intercept	0.19	0.06	0.07	0.30
μ	Question-Specificity (high)	0.67	0.10	0.47	0.87
μ	Disposition cooperative	-0.01	0.04	-0.10	0.07
μ	Disposition resistant	0.04	0.04	-0.03	0.12
μ	Design-Type	0.00	0.04	-0.07	0.08
μ	Question-Type:Design-Type	-0.03	0.03	-0.10	0.04
ϕ	Intercept	-1.68	0.02	-1.72	-1.64
ϕ	Question-Specificity	0.11	0.02	0.07	0.15
ϕ	Disposition cooperative	0.01	0.03	-0.04	0.06
ϕ	Disposition resistant	0.01	0.02	-0.04	0.06
ϕ	Design-Type	-0.02	0.02	-0.06	0.02

Table Z1: Population-level estimates of Model 1a in log-odds with the standard errors and 95% highest density intervals. Mean parameters are depicted first. The slope for Question-Specificity is the change in log-odds for the high-specificity question (1, high-specificity; −1, low-specificity), the slope for Disposition is the change in log-odds for cooperative and resistant participants (semi-cooperative was coded as -1, -1), and the slope for Design-Type is the change in log-odds for the within-subjects design (1, between, -1 within).

Differences between scenarios

Inspection of the distributions of response ratings across the six individual scenarios indicated that they were highly similar. This pattern held for both the within-subjects replication (Replication 1) and the between-subjects replication (Replication 2). Given the absence of meaningful distributional differences across scenarios, conducting separate statistical analyses at the scenario level would be redundant. For completeness, the corresponding distribution plots are provided in Figures Z1 (Replication 1) and Z2 (Replication 2).

Parameter	Coefficient	Posterior mean	Est. error	l-95% HDI	u-95% HDI
μ	Intercept	0.17	0.07	0.03	0.30
μ	Question-Specificity (high)	0.70	0.08	0.52	0.86
μ	Disposition (semi-cooperative)	-0.01	0.10	-0.20	0.18
μ	Disposition (resistant)	0.06	0.10	-0.13	0.24
ϕ	Intercept	-1.65	0.04	-1.73	-1.57
ϕ	Question-Specificity	0.11	0.03	0.06	0.16
ϕ	Disposition (semi-cooperative)	-0.02	0.06	-0.13	0.10
ϕ	Disposition (resistant)	-0.02	0.06	-0.12	0.09

Population-level estimates of Model 1a in log-odds with the standard errors and 95% highest density intervals. Mean parameters are depicted first. The slope for Question-Specificity is the change in log-odds for the high-specificity question (1, high-specificity; −1, low-specificity) and the slope for Disposition is the change in log-odds for resistant and semi-cooperative participants (cooperative was coded as the reference-level).

Parameter	Coefficient	Posterior mean	Est. error	l-95% HDI	u-95% HDI
μ	Intercept	0.17	0.10	-0.02	0.38
μ	Question-Specificity (high)	0.62	0.14	0.35	0.89
μ	Disposition (semi-cooperative)	-0.03	0.12	-0.26	0.20

μ	Disposition (resistant)	0.05	0.12	-0.18	0.28
ϕ	Intercept	-1.70	0.05	-1.81	-1.59
ϕ	Question-Specificity	0.11	0.03	0.05	0.17
ϕ	Disposition (semi-cooperative)	-0.03	0.07	-0.16	0.11
ϕ	Disposition (resistant)	0.03	0.07	-0.10	0.17

Population-level estimates of Model 2a in log-odds with the standard errors and 95% highest density intervals. Mean parameters are depicted first. The slope for Question-Specificity is the change in log-odds for the high-specificity question (1, high-specificity; −1, low-specificity) and the slope for Disposition is the change in log-odds for resistant and semi-cooperative participants (cooperative was coded as the reference-level).

Figure Z1

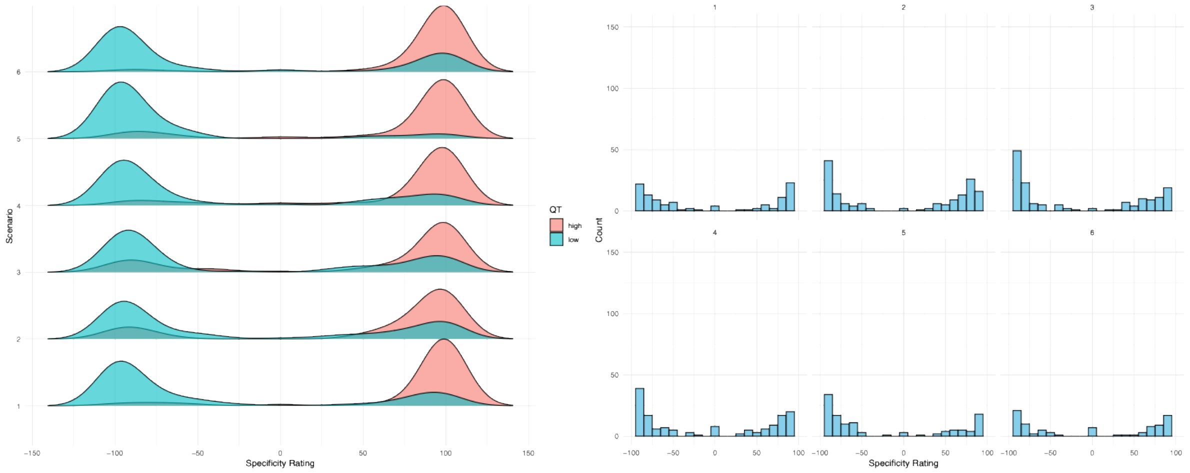

Figure Z1: Distribution of specificity ratings across the six scenarios for Replication 1. The left panel displays ridgeline density plots of response ratings by scenario, separated by question type (QT). The right panel shows faceted histograms (bin width = 10) of response ratings for each scenario. Across both visualizations, the distributions appear highly similar across scenarios, with pronounced bimodality at the scale endpoints (−100 and 100).

Figure Z2

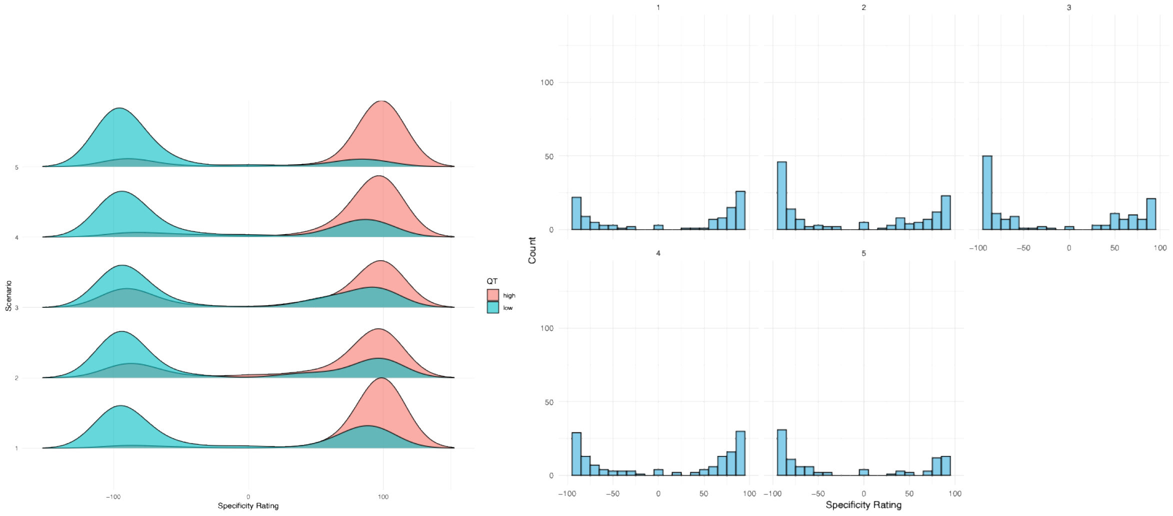

Figure Z2: Distribution of specificity ratings across the five scenarios for Replication 2. The left panel displays ridgeline density plots of response ratings by scenario, separated by question type (QT). The right panel shows faceted histograms (bin width = 10) of response ratings for each scenario. Across both visualizations, the distributions appear highly similar across scenarios, with pronounced bimodality at the scale endpoints (−100 and 100).

Appendix F

Replication 1: Wagers on Information Item designations

Participants also had the option to put a wager on how confident they were in identifying what the interviewer wanted to know. Figure X4 illustrates the participants' probability of betting for the different conditions.

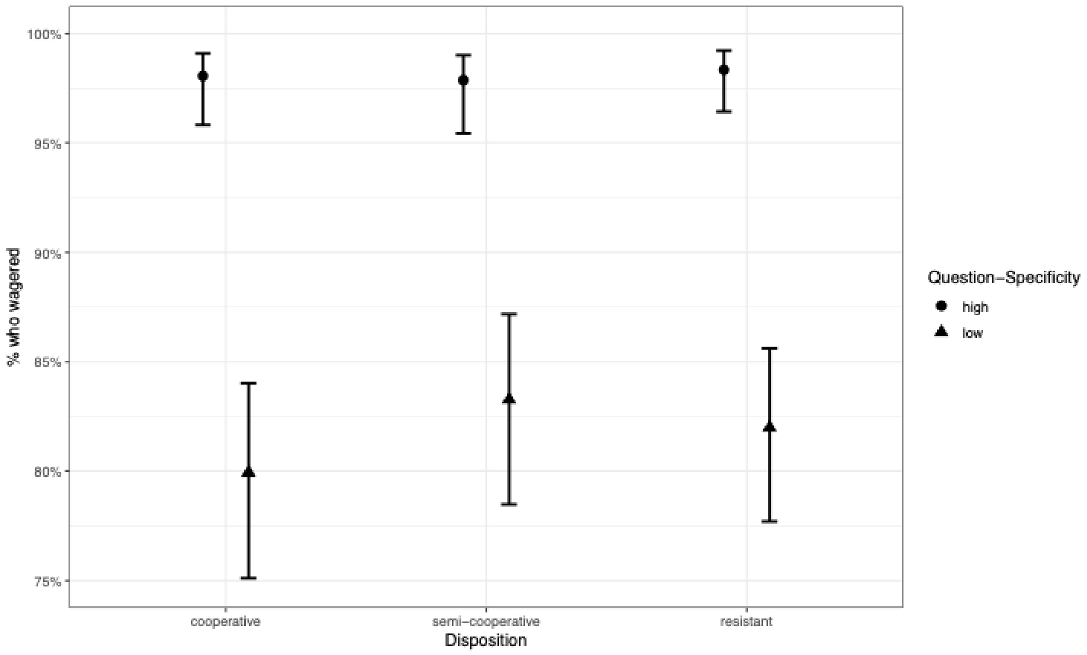

Figure X4: Proportion of participants who wagered as a function of disposition (cooperative, semi-cooperative, resistant) and question type (QT: low vs. high). Points represent observed proportions; error bars indicate 95% confidence intervals.

We quantified these observations using a logistic regression model. However, likely due to the pronounced ceiling effects in the data, the initial model exhibited sampling divergences. To address this issue and obtain stable estimates, we simplified the model by removing random slopes. None of the slope coefficients achieved the prespecified level of precision required to draw firm conclusions. Although the model suggests that high- as opposed to low-specificity questions were associated with a higher probability of wagering (b = 2.84, HDI: [2.31, 3.38]; see Table X3), this estimate should be interpreted with caution given the limited precision of the model.

**Coefficient**	**Posterior mean**	**Est. error**	**l-95% HDI**	**u-95% HDI**
Intercept	2.41	0.93	0.60	4.23
Question-Specificity (high)	2.84	0.27	2.31	3.38
Disposition (cooperative)	-0.09	0.30	-0.68	0.49
Disposition (resistant)	0.04	0.29	-0.52	0.61

Table X3: population-level estimates of Model 1c in log-odds with the standard errors and 95% highest density intervals. The slope for Question-Specificity is the change in log-odds for the high-specificity question (1, high-specificity; −1, low-specificity) and the slope for disposition is the change in log-odds for cooperative and resistant participants (semi-cooperative was coded as -1, -1).

Participants who chose to wager also were asked what percentage of their compensation they would bid on what the interviewer wants to know (0% = *none of my compensation*, 100% = *all of my compensation*). On average participants who placed a wager were willing to wager 72.6% of their compensation. Figure X5 illustrates differences between the low versus high question specificity. Across disposition levels participants seemed to wager more in the high as opposed to low Question-Specificity condition.

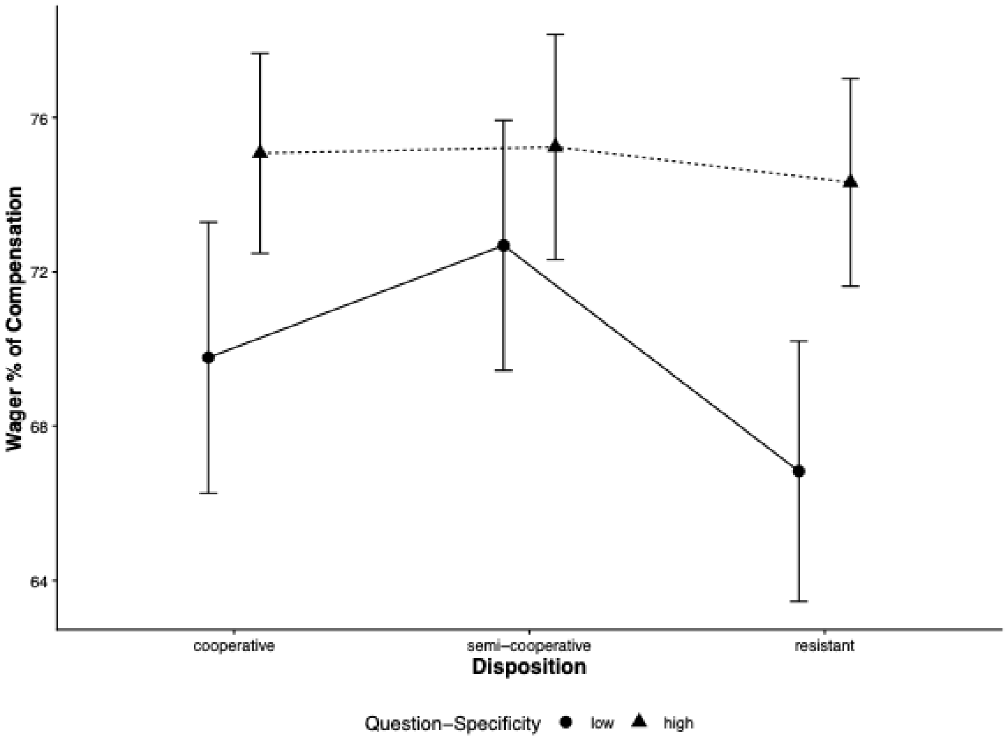

Figure X5: Participants' wager (% of their compensation) as a function of disposition (cooperative, semi-cooperative, resistant) and question type (QT: low vs. high). Points represent condition means; error bars indicate 95% confidence intervals.
